# Usefulness and Prognostic Impact of Preoperative Dynamic CT in the Diagnosis of Extrapancreatic Extension in Resectable Pancreatic Adenocarcinoma

**DOI:** 10.3390/cancers18111780

**Published:** 2026-05-29

**Authors:** Kazuma Horiguchi, Hiroyuki Kato, Takahiro Tashiro, Daisuke Koike, Hidetoshi Nagata, Yuka Kondo, Hironobu Yasuoka, Takashi Imanaka, Hiroki Tani, Yoshiki Kunimura, Masahiro Ito, Yutaro Kato, Tsunekazu Hanai, Zenichi Morise, Shuji Isaji, Ryota Hanaoka, Akihiko Horiguchi

**Affiliations:** 1 Department of Gastroenterological Surgery, Bantane Hospital, School of Medicine, Fujita Health University, 3-6-10 Otobashi, Nakagawa Ward, Nagoya 454-8509, Japan; 81023034@fujita-hu.ac.jp (K.H.); takahiro.tashiro@fujita-hu.ac.jp (T.T.); daisuke.koike@fujita-hu.ac.jp (D.K.); hnagata@fujita-hu.ac.jp (H.N.); yuccakon@fujita-hu.ac.jp (Y.K.); h.yasuoka@aichi-cc.jp (H.Y.); takashi.imanaka@fujita-hu.ac.jp (T.I.); hiroki.tani@fujita-hu.ac.jp (H.T.); yoshiki.kunimura@fujita-hu.ac.jp (Y.K.); masito@fujita-hu.ac.jp (M.I.); y-kato@fujita-hu.ac.jp (Y.K.); kouichi@fujita-hu.ac.jp (T.H.); zmorise@fujita-hu.ac.jp (Z.M.); akihori@fujita-hu.ac.jp (A.H.); 2 Department of Hepatobiliary Pancreatic and Transplant Surgery, Mie University, Tsu 514-8507, Japan; isaji-s@med.mie-u.ac.jp; 3 Department of Radiology, School of Medicine, Fujita Health University, Toyoake 470-1192, Japan; hanaoka@fujita-hu.ac.jp

**Keywords:** pancreatic ductal adenocarcinoma, extrapancreatic nerve plexus invasion, dynamic computed tomography

## Abstract

**Background/Objectives:** Pancreatic ductal adenocarcinoma (PDAC) remains one of the most lethal gastrointestinal cancers, and reliable preoperative predictors of aggressive tumor biology are essential for optimizing treatment strategies, particularly in resectable PDAC (RPDAC). This retrospective study evaluated the diagnostic accuracy of dynamic computed tomography (CT) for detecting extrapancreatic extension, peripancreatic plexus (PL), serosal (S), and retroperitoneal (RP) invasion, and assessed its prognostic significance. **Methods:** Ninety-four patients who underwent curative-intent upfront surgery for resectable PDAC between 2007 and 2020 were included. Dynamic CT was reviewed using standardized window settings (WL 35/WW 350) to identify soft-tissue projections extending beyond the pancreatic contour. **Results:** Pathological S, RP, and PL invasion occurred in 29.8%, 56.3%, and 17.0% of patients, respectively. Dynamic CT demonstrated accuracies of 73.4%, 76.6%, and 87.2% for S, RP, and PL invasion, respectively. Notably, patients with PL-positive CT findings had significantly poorer disease-specific survival (DSS) than those with PL-negative, with 3- and 5-year DSS rates of 37% and 0% versus 61% and 53% (*p* < 0.001). Multivariate analysis confirmed preoperative PL invasion as the only independent predictor of poor prognosis. **Conclusions:** Dynamic CT provides reasonable diagnostic performance for assessing extrapancreatic invasion. In addition, CT-identified PL invasion reflects aggressive tumor behavior and may justify consideration of neoadjuvant therapy, even in anatomically resectable disease.

## Simple Summary

Pancreatic cancer is one of the most aggressive cancers, and even patients whose tumors appear surgically removable often experience early recurrence. Identifying high-risk features before surgery is therefore crucial. In this study, we evaluated whether preoperative contrast-enhanced CT scans can detect tumor spread beyond the pancreas and predict prognosis in patients with resectable pancreatic cancer. We analyzed 94 patients who underwent upfront surgery and compared CT findings with pathological results and survival outcomes. CT showed good accuracy in detecting tumor extension outside the pancreas. Importantly, spread along the surrounding nerve plexus identified on CT was strongly associated with significantly worse survival. These findings suggest that specific CT findings may reflect aggressive tumor biology and could help guide treatment decisions, including consideration of preoperative therapy even when the tumor is technically resectable.

## 1. Introduction

Despite recent advances in systemic therapy, pancreatic ductal adenocarcinoma (PDAC) remains one of the most lethal gastrointestinal malignancies, and long-term survival is achieved only through surgical resection [1,2]. Early diagnosis of PDAC remains challenging [3,4], and most patients are diagnosed at an advanced stage. As many patients present with locoregional extension at the time of diagnosis, accurate assessment of resectability is essential for optimizing treatment strategies.

In recent years, PDAC resectability classification has been widely adopted in clinical practice [5]. According to the 2019 Japanese Clinical Practice Guidelines for Pancreatic Cancer, resectable PDAC (RPDAC) is defined as a tumor without portal venous involvement or with involvement of less than one-half of the vessel circumference [6]. The guidelines also recommend neoadjuvant chemotherapy with gemcitabine plus S-1, even for RPDAC [7]. In contrast, Western guidelines state that neoadjuvant therapy for RPDAC should generally be limited to clinical trials unless high-risk features, such as highly elevated carbohydrate antigen 19-9 (CA19-9), large primary tumors, markedly enlarged regional lymph nodes, excessive weight loss, or severe tumor-related pain, are present [8]. Thus, substantial discrepancies exist between Japan and Western countries regarding preoperative treatment strategies for RPDAC. One reason for this divergence is the lack of robust imaging-based criteria for reliably identifying patients who are likely to benefit from neoadjuvant therapy in the setting of RPDAC.

Pathological extrapancreatic extension, including peripancreatic plexus (PL), anterior serosal (S), and posterior retroperitoneal (RP) invasion, is frequently observed in pancreatoduodenectomy specimens and is strongly associated with poor prognosis [9,10,11]. Previous studies have investigated the radiological assessment of extrapancreatic extension [12,13]; however, variability in imaging protocols and lack of standardized CT window settings have limited the reproducibility and clinical applicability of these findings. Thus, preoperative diagnosis of extrapancreatic invasion remains challenging, and the diagnostic performance of dynamic computed tomography (CT) has not been fully established. Therefore, clarifying the accuracy of dynamic CT in predicting extrapancreatic extension is crucial for refining treatment strategies in patients with RPDAC. In the 8th edition of the Japanese Classification of Pancreatic Cancer [14], PL, S, and RP invasion are defined as specific patterns of extrapancreatic extension and are recognized as important pathological prognostic factors. Based on these definitions, the present study evaluated the clinical utility of preoperative dynamic CT for predicting pathological PL, S, and RP invasion under standardized imaging conditions. To the best of our knowledge, this is the first study to validate the CT-based diagnostic criteria of the Japanese Classification of Pancreatic Cancer for predicting pathological extrapancreatic extension under standardized CT conditions.

## 2. Materials and Methods

### 2.1. Study Design and Patient Selection

This retrospective study included patients who underwent upfront pancreatic resection for suspected pancreatic cancer at our institution. Between January 2007 and December 2020, 143 patients underwent surgical resection (RPDAC: 106, borderline resectable with portal vein involvement (BR-PV): 22, borderline resectable with arterial involvement (BR-A): 15). Among the 106 patients with clinically RPDAC, those with histopathologically confirmed invasive PDAC, excluding IPMC and neuroendocrine tumors, and with available preoperative dynamic CT imaging were selected. As a result, 94 patients who underwent upfront pancreatic resections were included in the final analysis. Surgical procedures comprised pancreatoduodenectomy (PD), distal pancreatectomy (DP), total pancreatectomy (TP), and other types of pancreatic resection. This study was approved by the Institutional Review Board of the hospital. Owing to the retrospective study design, informed consent was obtained using an opt-out approach in accordance with institutional and national ethical guidelines.

### 2.2. Preoperative Imaging Protocol

**Figure 1:**
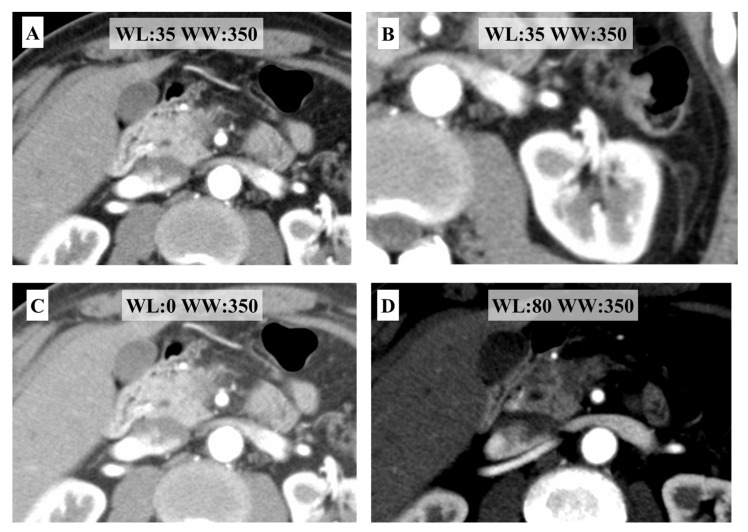
CT window settings for evaluation of extrapancreatic invasion. (**A**) WL 35/WW 350 (arterial phase). (**B**) Visualization of the Gerota fascia at WL 35/WW 350. (**C**) WL 0/WW 350 (overestimation). (**D**) WL 80/WW 350 (underestimation).

Preoperative imaging was performed using multidetector CT (MDCT). Dynamic contrast-enhanced CT was evaluated in two phases: the arterial and equilibrium phases. Because the detectability of extrapancreatic extension depends on window settings, images were interpreted using a standardized window level (WL) and window width (WW) setting of WL 35/WW 350, which provides optimal visualization of the Gerota fascia and retroperitoneal fat planes (Figure 1A,B). For reference, additional window settings shown in Figure 1 are as follows: WL 0/WW 350, allowing broader assessment of soft-tissue density (Figure 1C), and WL 80/WW 350, emphasizing pancreatic parenchymal contrast (Figure 1D).

### 2.3. Radiological Assessment of Extrapancreatic Extension

Radiological assessment of extrapancreatic extension was performed in accordance with the CT imaging guidelines of the 8th edition of the Japanese Classification of Pancreatic Cancer [14]. S and RP extrapancreatic invasion in pancreatic head cancer were defined as spicula-like projections extending beyond the anterior and posterior pancreatic surface into the surrounding peripancreatic adipose tissue, respectively. PL invasion was defined as club-shaped or cord-like soft-tissue attenuation continuous with the primary tumor, extending along the expected anatomical course of the nerve plexus toward the celiac artery or superior mesenteric artery, and observed on both arterial and equilibrium phase images. To minimize subjectivity, the assessment was performed according to predefined criteria under standardized CT window settings. Tumors showing direct invasion of or contact with the SMA or celiac artery were excluded, as such cases did not meet the definition of RPDAC. Diffuse or ill-defined inflammatory changes without clear continuity from the primary tumor were not considered indicative of plexus invasion. Moreover, lymphadenopathy was defined as lymph nodes with a short-axis diameter of ≥10 mm or those showing suspicious morphological features, including a round shape or heterogeneous attenuation on CT. CT images were interpreted in routine clinical practice by typically two board-certified radiologists. For the purpose of this study, the imaging findings were subsequently reviewed and confirmed by experienced hepatobiliary–pancreatic surgeons, who were blinded to pathological outcomes. In cases of discrepancy between readers, the findings were resolved by consensus through joint review.

### 2.4. Clinical Variables

Patient demographic and laboratory variables included age, sex, body mass index (BMI), serum albumin, hemoglobin, white blood cell count, prognostic nutritional index (PNI), neutrophil-to-lymphocyte ratio (NLR), CA19-9 level, and carcinoembryonic antigen (CEA) level. Tumor-related factors included tumor location (pancreatic head vs. body or tail) and tumor size measured on preoperative CT. Preoperative CT findings included portal venous invasion, common hepatic duct (CH) invasion, duodenal (DU) invasion, S invasion on CT, RP invasion on CT, PL invasion on CT, and lymphadenopathy, each recorded as present or absent. Surgical factors analyzed included the type of surgical procedure performed (pancreatoduodenectomy, distal pancreatectomy, total pancreatectomy, or other procedures), operative time, and intraoperative blood loss. All analyses were designed to evaluate peri-operative clinical and radiological predictors of prognosis, including selected pathological variables corresponding to imaging findings. Therefore, intraoperative findings, postoperative complications, and adjuvant therapy were not included in the present analysis.

### 2.5. Statistical Analysis

The primary endpoint was disease-specific survival (DSS). Survival curves were generated using the Kaplan–Meier method, and prognostic factors associated with DSS were evaluated using the log-rank test for univariate analysis. The follow-up period was estimated using the reverse Kaplan–Meier method. Variables with a *p*-value < 0.1 in univariate analysis were subsequently included in multivariate analysis using the Cox proportional hazards model. The proportional hazards assumption was assessed using log-minus-log survival plots. Multicollinearity among variables was evaluated using variance inflation factors. All continuous variables (age, BMI, albumin, hemoglobin, CA19-9, and others) were analyzed as continuous variables and were not dichotomized. A two-sided *p* value < 0.05 was considered statistically significant. All statistical analyses were performed using SPSS Statistics version 28 (IBM Corp., Armonk, NY, USA).

## 3. Results

### 3.1. Patient Characteristics

The clinical characteristics of the 94 patients included in this study are summarized in Table 1. The median age of the patients was 70 years (range, 43–88 years), and the cohort consisted of 48 males and 46 females. The median BMI was 21.2 kg/m^2^ (range, 14–38 kg/m^2^), albumin was 4.1 g/dL (range, 2.9–5.2 g/dL), hemoglobin was 12.9 g/dL (range, 6.5–15.9 g/dL), and white blood cell count was 5700/mm^3^ (range, 2500–12,500/mm^3^). The median PNI and NLR were 47.6 (range, 33.1–64.0) and 2.8 (range, 0.64–17.8), respectively.

Median tumor marker levels were 96.7 U/L (range, 0.1–15,950 U/L) for CA19-9 and 3.2 U/L (range, 0.7–99.7 U/L) for CEA. Tumors were located in the pancreatic head, body, and tail in 81, 7, and 6 patients, respectively. The median tumor size measured on CT was 25 mm (range, 4–63 mm).

### 3.2. Preoperative CT Findings and Surgical Outcomes

**Figure 2:**
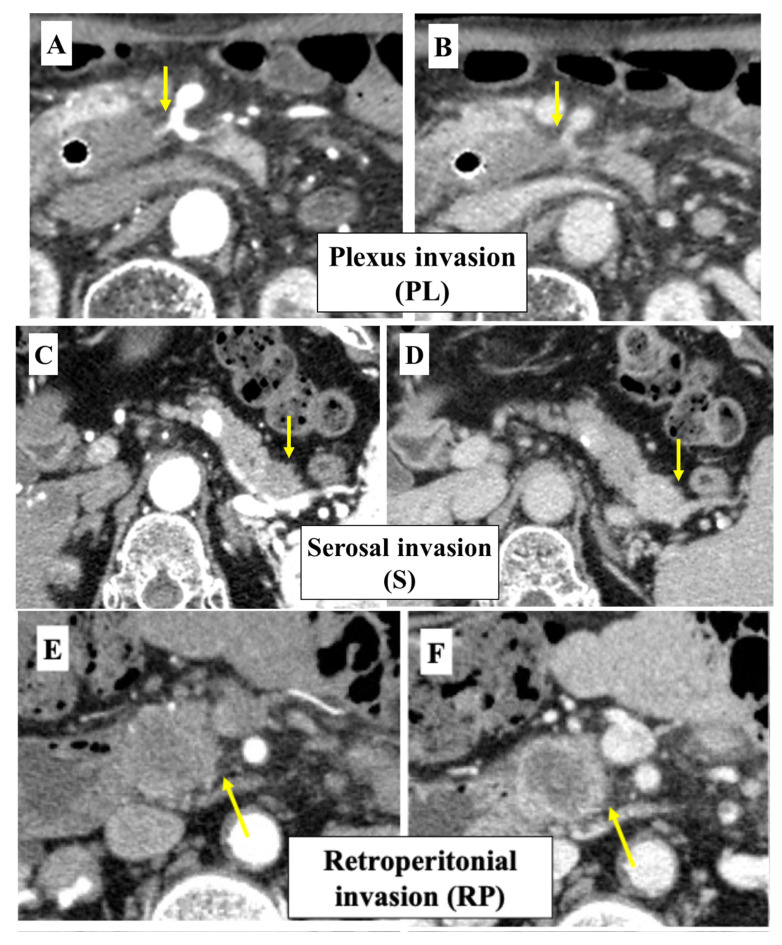
Representative CT findings of extrapancreatic invasion. (**A**,**B**) PL invasion (arterial and equilibrium phases). (**C**,**D**) S invasion (arterial and equilibrium phases). (**E**,**F**) RP invasion (arterial and equilibrium phases).

Preoperative CT findings revealed portal vein invasion in 24.5% of patients (23/94), S invasion in 45.7% (43/94), RP invasion in 50.0% (47/94), PL invasion in 14.9% (14/94), and lymphadenopathy in 48.9% (46/94) (Figure 2). Surgical procedures included 82 pancreatoduodenectomies, 11 distal pancreatectomies, and 1 other pancreatic resection. The median operative time was 428 min (range, 145–809 min), and the median operative blood loss was 403 g (range, 23–2109 g). An R0 resection was achieved in 86.2% of patients (81/94).

### 3.3. Pathological Findings and Diagnostic Performance of Dynamic CT

**Figure 3:**
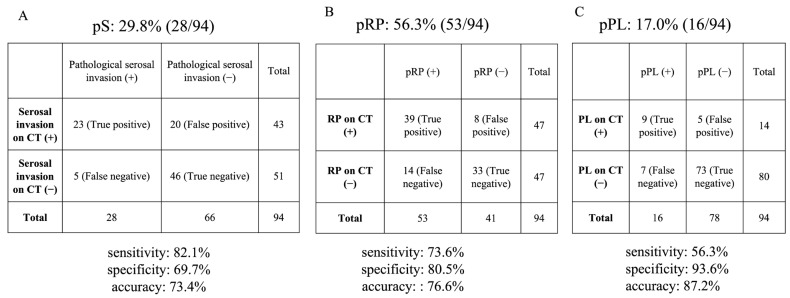
Concordance between preoperative CT findings and pathological extrapancreatic extension. (**A**) Concordance between serosal invasion on CT and pathological serosal invasion (pS). (**B**) Concordance between RP on CT and pathological RP (pRP). (**C**) Concordance between PL on CT and pathological PL (pPL). Each panel shows contingency tables indicating true-positive, false-positive, false-negative, and true-negative cases. Sensitivity, specificity, and accuracy were calculated based on these contingency tables.

Pathological extrapancreatic extension was frequently observed in the resected specimens. The incidence of pathological serosal invasion (pS), pathological retroperitoneal invasion (pRP), and pathological plexus invasion (pPL) was 29.8% (28/94), 56.3% (53/94), and 17.0% (16/94), respectively (Figure 3). The diagnostic performance of preoperative dynamic CT in detecting pathological extrapancreatic extension varied according to the type of invasion. For pS, sensitivity, specificity, and accuracy were 82.1%, 69.7%, and 73.4%, respectively. The corresponding values for pRP were 73.6%, 80.5%, and 76.6%, respectively. For pPL, sensitivity, specificity, and accuracy were 56.3%, 93.6%, and 87.2%, respectively.

### 3.4. Survival Analysis and Prognostic Factors

**Figure 4:**
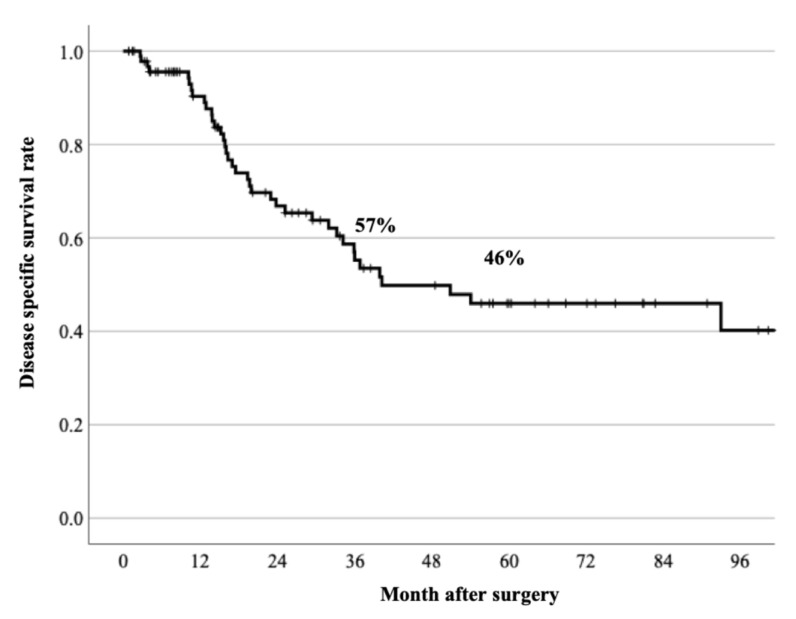
Disease-specific survival of all 94 patients.

**Figure 5:**
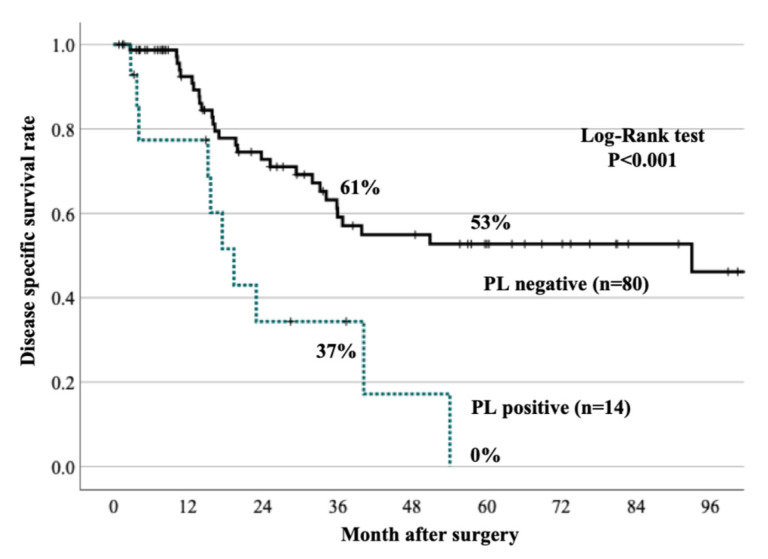
Disease-specific survival according to the presence or absence of plexus invasion (PL) on preoperative CT.

DSS for all 94 patients is shown in Figure 4. The 3- and 5-year DSS rates were 57% and 46%, respectively. The median follow-up period was 57.5 months. DSS differed markedly according to the presence or absence of plexus invasion on preoperative CT (Figure 5). Patients with PL-positive CT findings (n = 14) had significantly poorer DSS than those without PL (n = 80), with a 3-year DSS rate of 37% versus 61%, respectively. The 5-year DSS rates in the PL-positive and negative groups were 0% and 53%, respectively. (log-rank test, *p* < 0.001). In univariate analysis (Table 2), preoperative PL on CT (*p* = 0.004) and longer operative time (*p* = 0.013), pathological PV invasion (*p* = 0.049) and pathological PL invasion (*p* = 0.047) were associated with unfavorable DSS. Furthermore, in multivariate analysis (Table 3), preoperative PL emerged as an independent prognostic factor for DSS (*p* = 0.026, events-per-variable ratio:8.2). Taken together, dynamic CT using the present imaging settings demonstrated reasonable diagnostic accuracy for extrapancreatic extension, particularly for PL invasion. Importantly, preoperative PL identified on CT was a strong and independent predictor of poor prognosis in patients undergoing pancreatic resection.

## 4. Discussion

In the present study, the clinical significance of three radiological patterns of extrapancreatic extension, S, RP, and PL invasion, was evaluated in patients with RPDAC who underwent upfront surgery. The results demonstrated that only preoperative PL was a significant predictor of poor postoperative survival, whereas S and RP were not associated with prognosis. These findings indicate that radiological nerve plexus invasion represents a distinct and clinically relevant feature that cannot be substituted by other forms of extrapancreatic extension.

Previous studies have demonstrated the prognostic superiority of radiologically assessed extrapancreatic extension over pathological evaluation in PDAC. A representative study by Toshima et al. [13] analyzed resected PDAC cases and reported that CT-diagnosed extrapancreatic extension was significantly associated with poor disease-free survival (hazard ratio, 4.22; *p* < 0.01) and overall survival (hazard ratio, 4.38; *p* < 0.01), whereas pathologically diagnosed extension failed to retain independent prognostic significance in multivariate analysis. These findings suggest that radiological tumor spread reflects the biological aggressiveness of PDAC more accurately than postoperative pathological assessment.

Consistent with this concept, the present study further refines the clinical relevance of radiological evaluation by demonstrating that, among distinct patterns of extrapancreatic extension, only nerve plexus invasion identified on preoperative CT independently predicts DSS in patients with RPDAC undergoing upfront curative-intent resection. In clinical practice, PL-positive findings on CT are often regarded as a surrogate marker of borderline resectable arterial disease. However, according to formal definitions provided by the National Comprehensive Cancer Network [8] and the International Association of Pancreatology guidelines [5], borderline resectable arterial involvement is characterized by direct tumor contact with the SMA < 180°. Tumors showing extension toward the inferior pancreaticoduodenal artery or the first jejunal artery, with preservation of the SMA circumference, should still be classified as RPDAC. Consequently, such patients have frequently been selected for an upfront surgery–first approach [15]. The present results indicate that even within this anatomically resectable population, PL positivity identifies a subgroup with clearly inferior outcomes, suggesting that current resectability criteria may underestimate tumor aggressiveness.

A major difficulty in evaluating PL preoperatively is the lack of a reliable and reproducible imaging-based assessment method. In this study, PL was assessed using radiological criteria based on the Japanese Pancreatic Cancer Classification with a standardized interpretation of dynamic CT images, allowing pathological PL to be predicted preoperatively with relatively high accuracy. Although inflammatory changes may mimic plexus invasion on CT, the requirement for continuous tumor extension along the anatomical course of the nerve plexus and the observed association with pathological findings and survival outcomes support the diagnostic validity of our criteria. To our knowledge, no previous reports have evaluated extrapancreatic nerve plexus invasion under unified CT window conditions with explicit diagnostic criteria. These findings are consistent with previous studies showing that tumor spreading into the plexus pancreaticus capitalis reflect an aggressive biological phenotype and often precede overt arterial invasion. Sugimoto et al. demonstrated that extrapancreatic nerve plexus invasion on CT was the only imaging factor independently associated with DSS and recurrence-free survival in anatomically resectable pancreatic cancer [16]. Furthermore, previous studies suggest that perineural invasion in PDAC reflects both aggressive tumor biology and advanced local tumor extension. Experimental studies have shown that tumor–nerve interactions promote tumor progression, supporting its role as a feature of tumor aggressiveness [17,18]. Conversely, clinicopathological studies indicate that nerve plexus invasion often coexists with retroperitoneal infiltration, suggesting that it may also represent local tumor extent [19]. Consistent with prior radiological studies, including Sugimoto et al. [16], CT-defined plexus invasion is associated with poor survival, although its interpretation as a distinct biological marker remains controversial. Overall, radiological PL invasion should be regarded as a composite indicator reflecting both tumor extent and biological aggressiveness rather than a purely independent biomarker.

From an anatomical perspective, the neural plexus surrounding the SMA and celiac axis represents a critical route of subclinical local tumor extension. Continuous soft-tissue strands or reticular densities extending toward these structures on dynamic CT, as described in the Japanese classification system, should therefore be regarded as radiological findings suggestive of early neural invasion. Several mechanisms may explain the poor prognosis observed in PL-positive patients, including facilitation of tumor–nerve interactions [17,18] within the perineural microenvironment that enables longitudinal tumor spread and microscopic residual disease despite macroscopic curative resection, frequent coexistence of nerve plexus invasion with retroperitoneal infiltration [20], and the possibility that neural invasion serves as a crucial marker of biologically aggressive disease characterized by enhanced stromal interaction, neurotrophic signaling, and early systemic dissemination [19,21]. On the other hand, the lack of prognostic significance of S and RP invasion, despite their relatively high pathological incidence, may be explained by the fact that these patterns primarily reflect local tumor extension rather than biological aggressiveness.

These findings have important clinical implications. Even in patients classified as anatomically resectable, radiological evidence of PL may warrant reconsideration of an upfront surgery–first strategy, particularly in light of recent randomized trials demonstrating that neoadjuvant treatment improves R0 resection rates and disease-free survival in resectable or borderline resectable pancreatic cancer [22,23]. Incorporation of PL into preoperative risk stratification may help identify patients who are unlikely to benefit from immediate surgery. However, further prospective studies are required to validate these results.

## 5. Conclusions

The present study is the first to demonstrate that radiological assessment of extrapancreatic nerve plexus invasion can be reliably performed by evaluating dynamic CT images under window settings optimized for visualization of the Gerota fascia. In addition, this study validates the CT-based diagnostic criteria for plexus invasion proposed in the 8th edition of the Japanese Pancreatic Cancer Classification [14]. Using this standardized imaging approach, preoperative plexus invasion was accurately predicted and identified as the sole extrapancreatic extension factor associated with a poor prognosis in patients with RPDAC undergoing upfront surgery. These findings indicate that radiological plexus invasion reflects aggressive tumor biology beyond conventional anatomical resectability criteria and may serve as a practical imaging biomarker for risk stratification. Incorporation of this CT-based evaluation into routine preoperative assessment may facilitate more appropriate treatment selection, including consideration of neoadjuvant therapy, even in patients with anatomically resectable disease.

## Figures and Tables

**Table 1:** Baseline patient characteristics.

Variable	Value (n = 94)
Age (years)	70 (43–88)
Sex (male/female)	48/46
BMI (kg/m^2^)	21.2 (14–38)
Serum albumin (g/dL)	4.1 (2.9–5.2)
Hemoglobin (g/dL)	12.9 (6.5–15.9)
White blood cell count (/mm^3^)	5700 (2500–12,500)
PNI	47.6 (33.1–64.0)
NLR	2.8 (0.64–17.8)
CA19-9 (U/mL)	96.7 (0.1–15,950)
CEA (U/mL)	3.2 (0.7–99.7)
Tumor location (head/body/tail)	81/7/6
Tumor size on CT (mm)	25 (4–63)
Portal vein invasion on CT (%)	24.5 (23/94)
S (%)	45.7 (43/94)
RP (%)	50.0 (47/94)
PL (%)	14.9 (14/94)
Lymphadenopathy (%)	48.9 (46/94)
Type of procedure (PD/DP/others)	82/11/1
Operation time (min)	428 (145–809)
Blood loss (g)	403 (23–2109)
R0 resection rate (%)	86.2 (81/94)

BMI, body mass index; CA19-9, carbohydrate antigen 19-9; CEA, carcinoembryonic antigen; CT, computed tomography; DP, distal pancreatectomy; NLR, neutrophil-to-lymphocyte ratio; PD, pancreatoduodenectomy; S, Serosal invasion, RP, retroperitoneal invasion; PL, plexus invasion; PNI, prognostic nutritional index; R0, microscopically margin-negative resection; Values in parentheses indicate the range (minimum–maximum) for continuous variables and the number of positive cases over the total number of patients (n/N) for categorical variables.

**Table 2:** Univariate analysis for predicting prognosis.

Perioperative Prognostic Factors	Hazard Ratio	95% CI	*p* Value
Age	1.017	0.976–1.058	0.424
Sex (female vs. male)	1.106	0.584–2.092	0.757
BMI (kg/m^2^)	0.939	0.853–1.034	0.200
Albumin (g/dL)	1.492	0.683–3.256	0.315
Hemoglobin (g/dL)	1.030	0.857–1.238	0.751
White blood cell count (/mm^3^)	1.000	1.000–1.000	0.730
Prognostic nutrition index (PNI)	0.998	0.945–1.054	0.933
NLR (neutrophil/lymphocyte ratio)	1.035	0.924–1.158	0.553
CA19-9 level (U/L)	1.000	1.000–1.000	0.677
CEA level (U/L)	1.013	0.992–1.035	0.230
Tumor location (head vs. others)	0.501	0.154–1.631	0.251
Tumor size on CT (mm)	1.012	0.987–1.037	0.340
Portal venous invasion on CT (no vs. yes)	1.547	0.681–3.515	0.298
CH on CT (yes vs. no)	1.267	0.638–2.519	0.499
DU on CT (yes vs. no)	1.241	0.656–2.347	0.507
S on CT (yes vs. no)	1.307	0.675–2.532	0.426
RP on CT (yes vs. no)	1.488	0.779–2.841	0.228
**PL on CT (yes vs. no)**	**2.950**	**1.** **414** **–** **6.135**	**0.004**
Lymphadenopathy on CT (yes vs. no)	1.565	0.816–3.003	0.178
Type of procedures (PD vs. others)	1.679	0.515–5.470	0.390
Operation time (min)	**1.004**	**1.001–1.006**	**0.013**
Blood loss (g)	1.000	1.000–1.000	0.506
**pPV** (yes vs. no)	**2.155**	**1.004–** **4** **.630**	**0.049**
pCH (yes vs. no)	1.330	0.678–2.604	0.407
pDU (yes vs. no)	1.277	0.664–2.458	0.465
pS (yes vs. no)	0.794	0.375–1.681	0.545
**pRP** (yes vs. no)	**1.946**	**0.993**–**3.817**	**0.053**
**pPL** (yes vs. no)	**2.041**	**1.010**–**4.115**	**0.047**

BMI, body mass index; CA19-9, carbohydrate antigen 19-9; CEA, carcinoembryonic antigen; CH, common hepatic duct invasion; CI, confidence interval; CT, computed tomography; DU, duodenum invasion; DP, distal pancreatectomy; NLR, neutrophil-to-lymphocyte ratio; PD, pancreaticoduodenectomy; PL, plexus invasion; PNI, prognostic nutritional index; RP, retroperitoneum invasion; S, Serosal invasion.

**Table 3:** Multivariate analysis for predicting prognosis.

Patient Backgrounds (n = 94)	Hazard Ratio	95% CI	*p*-Value
Operation time (min)	1.002	0.998–1.005	0.388
PL on CT (yes vs. no)	2.577	1.119–5.917	0.026
pPV (yes vs. no)	1.709	0.728–4.016	0.218
RP on CT (yes vs. no)	1.462	0.677–3.155	0.333
pPL (yes vs. no)	1.225	0.496–3.025	0.661

CI: confidence interval. CT: computed tomography. PL: plexus invasion. pPL: pathological plexus invasion. pPV: pathological portal vein invasion. RP: retroperitoneal invasion.

## Data Availability

The data presented in this study are available from the corresponding author upon reasonable request. The data are not publicly available due to privacy and ethical restrictions.

## Abbreviations

The following abbreviations are used in this manuscript:

BMI	Body mass index
CA19-9	Carbohydrate antigen 19-9
CEA	Carcinoembryonic antigen
CI	Confidence interval
CT	Computed tomography
DSS	Disease-specific survival
DP	Distal pancreatectomy
DU	Duodenal invasion
MDCT	Multidetector computed tomography
NLR	Neutrophil-to-lymphocyte ratio
PD	Pancreaticoduodenectomy
PDAC	Pancreatic ductal adenocarcinoma
PL	Extrapancreatic nerve plexus invasion
PNI	Prognostic nutritional index
RP	Retroperitoneal invasion
RPDAC	Resectable pancreatic ductal adenocarcinoma
R0	Microscopically margin-negative resection
S	Serosal invasion
SMA	Superior mesenteric artery
TP	Total pancreatectomy
WBC	White blood cell count
WL	Window level
WW	Window width
CH	Common hepatic duct invasion
pPL	Pathological plexus invasion
pRP	Pathological retroperitoneal invasion
pS	Pathological serosal invasion
